# A study of allergen detection panel in Guangzhou, southern China based on real-world data from the past 7 years

**DOI:** 10.1038/s41598-023-41949-x

**Published:** 2023-09-08

**Authors:** Haisheng Hu, Huimin Huang, Chenxi Liao, Aoli Li, Teng Zhang, Xueqing Liang, Baoqing Sun

**Affiliations:** 1 Department of Clinical Laboratory, National Center for Respiratory Medicine, National Clinical Research Center for Respiratory Disease, State Key Laboratory of Respiratory Disease, Guangzhou Institute of Respiratory Health, The First Affiliated Hospital of Guangzhou Medical University, 151 Yanjiangxi, Guangzhou, China; 2 Department of Clinical Laboratory, The People’s Hospital of Zhuhai, Zhuhai, China; 3 China Institute for Radiation Protection, Taiyuan, China

**Keywords:** Diagnosis, Population screening

## Abstract

This study aims to reduce the cost of allergen testing for Guangzhou, China by limiting the number of allergens for which patients are tested, and provide a testing panel to improve diagnostic and therapeutic efficiency. This retrospective study of real-world data from 2012 to 2019 included 39,570 patients with suspected allergies in Guangzhou, southern China. All the patients were tested for one or more of the following allergens serum specific immunoglobulin E (sIgE): *Dermatophagoides pteronyssinus*, *Dermatophagoides farinae*, cat dander, dog dander, *Artemisia vulgaris*, *Aspergillus fumigatus*, *Alternaria alternata*, *Blattella germanica*, egg whites, milk, wheat, peanuts, soybeans, *Cancer pagurus*, and *Penaeus monodon* by PhadiaCAP 1000. Totally, only the positive rates of allergens sIgE in *D. farinae*, *D. pteronyssinus*, milk, egg whites, *B. germanica*, *C. pagurus*, *A. alternata*, and *P. monodon* were > 10%, the other allergens were between 4–7%. Moreover, among the allergic diseases, dust mites exhibited the overall highest positive rate, followed by milk and *B. germanica*. In children, milk was the main allergen, whereas in adults, mites, cockroaches, shrimp, and crab allergens had higher positive rates. The optimal scale analysis shows that the multiple sensitization classification of patients can be divided into three categories: I *D. farinae* and *D. pteronyssinus*; II. *C. pagurus*, *P. monodon*, and *B. germanica*; III. Milk and egg whites. Generally, a panel including 4 allergens can detect > 90% of the potential allergy in this local population. In Guangzhou, southern China, *D. farinae*, milk, *B. germanica*, and *A. alternata* as a panel screening allergy for suspected allergic patients was suggested base on this study.

## Introduction

In recent years, allergy has become one of the major causes of chronic respiratory diseases in the world, with a incidence rate of more than 27%, posing a heavy medical burden on patients and their families^1,2^. Allergens play an important role in allergic reactions. In southern China, the types of allergens can reach thousands, and the sensitization situation has been evolving with the shift in people's lifestyles and diet structure^3^. A patient with potential allergy who is unaware of the allergen he has been exposed to, making it difficult for the doctor to rule out allergens and select which ones to test for. Therefore, some hospitals will develop a panel for allergen diagnosis, which may contain 4–8 allergens (inhaled and ingested allergens may be develop on the same panel sometimes)^4^. Clinicians can select different test panels depending on the patient’s age, region, and living habits. Allergens contained in these panels are generally common in the region. However, not all local common allergens can be detected, because it depends on the allergen detection reagents available in the Chinease market^5^. In Guangzhou, southern China, a total of 15 allergens had been included in the checklist in the past 7 years, of which, eight are inhaled and seven are ingested. This study aimed to explore how doctors select allergens to be tested and the positive distribution of these allergens in the past 7 years, to assess current diagnostic strategies and provide support for development of an allergen detection panel in this region.

## Methods

### Study design

As a retrospective study, real-world data was extracted from the National Clinical Research Center for Respiratory Disease (BRD-NCRCRD, Guangzhou, China) for patients with suspected allergies from January 1, 2012 to December 31, 2019. All the patients were suspected for having allergy by doctors based on symptoms, such as dyspnea, runny nose, sneezing, nasal itching/obstruction, rashes, wheal, urticaria, abdominal pain, diarrhea, indigestion, and itchy eyes. Then, serum specific immunoglobulin E tests conducted using PhadiaCAP (ThermoFisher, USA) were used to confirm allergen(s) causing sensitization. Patients with cancer, immunodeficiency, parasitic infection, autoimmune diseases, and those with no data regarding age were excluded, and the remaining patients were enrolled in this study.

### Detection method

In all the patients, 5 mL of venous blood samples was extracted, separated using gel vacuum coagulation tube, and centrifuged at 3000 rpm for 10 min. The upper serum was collected to detect the following allergens: *Dermatophagoides pteronyssinus* (House dust mite), *Dermatophagoides farinae* (House dust mite), cat dander, dog dander, *Artemisia vulgaris* (Mugwort), *Aspergillus fumigatus* (fungus), *Alternaria alternata* (fungus), *Blattella germanica* (cockroach), egg whites, milk, wheat, peanuts, soybeans, *Cancer pagurus* (Crab), and *Penaeus monodon* (shrimp). Detection the serum sIgE using PhadiaCAP 1000 (ThermoFisher, USA) was performed by trained technicians, and the results are presented as kU/L, with ≥ 0.35 kU/L as the positive cutoff value.

### Statistical analysis

Data were analyzed using the statistical software package SPSS 22.0 (Chicago, IL, USA). To represent the proportion of positive results, categorical data are reported as percentages. Chi-square (χ^2^) tests or F-tests were used to demonstrate differences in proportions between groups. Calculate the correlation coefficient between two allergen sIgE concentrations using Spearman correlation analysis, denoted by *r*_*s*_. The optimal scale analyses were performed to identify the connection and classification between multiple allergens sIgE. Statistical significance was set at *P* < 0.05.

### Ethics approval and informed consent

Approval was obtained from the Ethics Committee of The First Affiliated Hospital of Guangzhou Medical University (reference no. GYFYY-2016-73). All methods were performed in accordance with the relevant guidelines and regulations. The informed consent of patients was obtained by Biobank for Respiratory Diseases in the National Clinical Research Center for Respiratory Disease (BRD-NCRCRD) (Guangzhou, Southern China), which informed each patient that their clinical examination data would be used for possible future studies.

## Results

### Sensitization of patients with different characteristics

Altogether, 39,570 patients were enrolled in the study; 8395 of whom had been diagnosed before 2012 (21.2%), and a majority of whom had been diagnosed in 2018 (25.4%). Of the patients, 45.3% were < 14 years old, and only 11.3% were > 65 years old. Interestingly, asthma or bronchitis (61.9%) had the highest prevalence among the patients (Table S1). Moreover, the highest rate of sensitization in the patients was to *D. farinae* (60.1–78.8%), followed by *D. pteronyssinus* (47.7–55.1%), and milk (32.1–35.8%). The top five allergens with the highest positive rate were *D. farinae*, *D. pteronyssinus*, milk, *A. alternate*, and egg whites in 2015 and 2016 and *D. farinae*, *D. pteronyssinus*, milk, egg whites, and *C. pagurus* in 2017 and 2019*.* For inhaled allergens, the top three allergens with the highest positive rate were *D. farinae*, *D. pteronyssinus*, and *A. alternata* in 2015 and 2016 and *D. farinae*, *D. pteronyssinus*, and *B. germanica* after 2016*.* For food allergens, the top three allergens with the highest positive rate were egg whites, milk, and *P. monodon* in 2015 and 2016 and egg whites, milk, and *C. pagurus* after 2016*.* Additionally, the top three inhaled allergens with the highest positive rate were *D. farinae*, *D. pteronyssinus*, and *B. germanica* in patients with asthma, allergic dermatitis, chronic obstructive pulmonary disease (COPD), and chronic cough and *D. farinae*, *D. pteronyssinus*, and *A. alternata* in those with rhinitis and bronchitis*.* For food allergens, the top three allergens with the highest positive rate were milk, egg whites, and *P. monodon* in patients with the abovementioned diseases, except for those with COPD with *P. monodon*, *C. pagurus*, and peanuts as their main allergens. In patients aged < 5 years, milk had the highest positive rate, although after 5 years, *D. farinae* became the top allergen. Further details are presented in Table 1.

**Table 1:** Top five allergens with the highest positive rate in different classifications.

Classification	Allergen with the top five positive rates (n, %)				
Year					
≤ 2015	**d2**	**d1**	**f2**	**m6**	**f1**
	739, 78.8%	1478, 55.1%	326, 32.2%	55, 26.4%	230, 23.3%
2016	**d2**	**d1**	**f2**	**m6**	**f1**
	485, 72.9%	1271,49.4%	599, 35.8%	30, 28.8%	456, 27.6%
2017	**d2**	**d1**	**f2**	**f1**	**f23**
	710, 60.9%	1642,47.9%	857, 33.5%	614, 24.1%	106, 20.9%
2018	**d2**	**d1**	**f2**	**f1**	**i6**
	1072, 61.3%	2034, 47.7%	1147, 35.3%	812, 25.2%	680, 17.8%
2019	**d2**	**d1**	**f2**	**f23**	**f1**
	750, 64.4%	1463, 49.0%	740, 32.1%	43, 22.8%	521, 22.2%
Disease					
Asthma	**d2**	**d1**	**f2**	**i6**	**f1**
	1661, 77.8%	2980, 61.2%	673, 30.1%	1011, 24.7%	550, 24.6%
Rhinitis	**d2**	**d1**	**f2**	**m6**	**f1**
	533, 77.0%	1070, 66.8%	262, 27.4%	6, 24.0%	212, 22.4%
Allergic dermatitis	**d1**	**d2**	**f2**	**f23**	**f1**
	304, 47.9%	258, 46.7%	145, 33.8%	48, 29.4%	110, 25.8%
COPD	**d2**	**d1**	**i6**	**f24**	**m3**
	24, 40.7%	80, 20.1%	65, 16.6%	51, 14.7%	94, 13.6%
Chronic cough	**d2**	**d1**	**f2**	**f1**	**i6**
	229, 59.6%	557, 44.8%	236, 29.5%	182, 22.6%	216, 19.2%
Infected	**d2**	**d1**	**f2**	**f1**	**m6**
	845, 57.3%	2351, 40.3%	2058, 37.6%	1383, 25.5%	34, 14.7%
Other	**d2**	**d1**	**f2**	**m6**	**f1**
	206, 53.9%	546, 40.5%	294, 33.3%	49, 28.2%	196, 22.2%
Age					
0–4 years	**f2**	**d2**	**d1**	**f1**	**m6**
	2994, 45.7%	554, 44.3%	1929, 34.2%	1963, 30.3%	6, 9.2%
5–14 years	**d2**	**d1**	**f23**	**i6**	**m6**
	1925, 83.5%	3712, 74.0%	110, 27.3%	996, 23.4%	28, 21.4%
15–44 years	**d2**	**d1**	**i6**	**f24**	**f23**
	883, 68.7%	1380, 57.6%	686, 35.2%	468, 28.6%	205, 25.0%
45–64 years	**d2**	**d1**	**i6**	**f24**	**f23**
	266, 46.1%	577, 31.8%	388, 23.6%	276, 18.9%	120, 16.4%
> 65 years	**d2**	**d1**	**i6**	**f24**	**f23**
	70, 42.9%	203, 23.9%	136, 16.7%	111, 15.6%	35, 12.6%

*COPD* chronic obstructive pulmonary disease.

### Multiple sensitization and classification in sensitized patients

**Figure 1:**
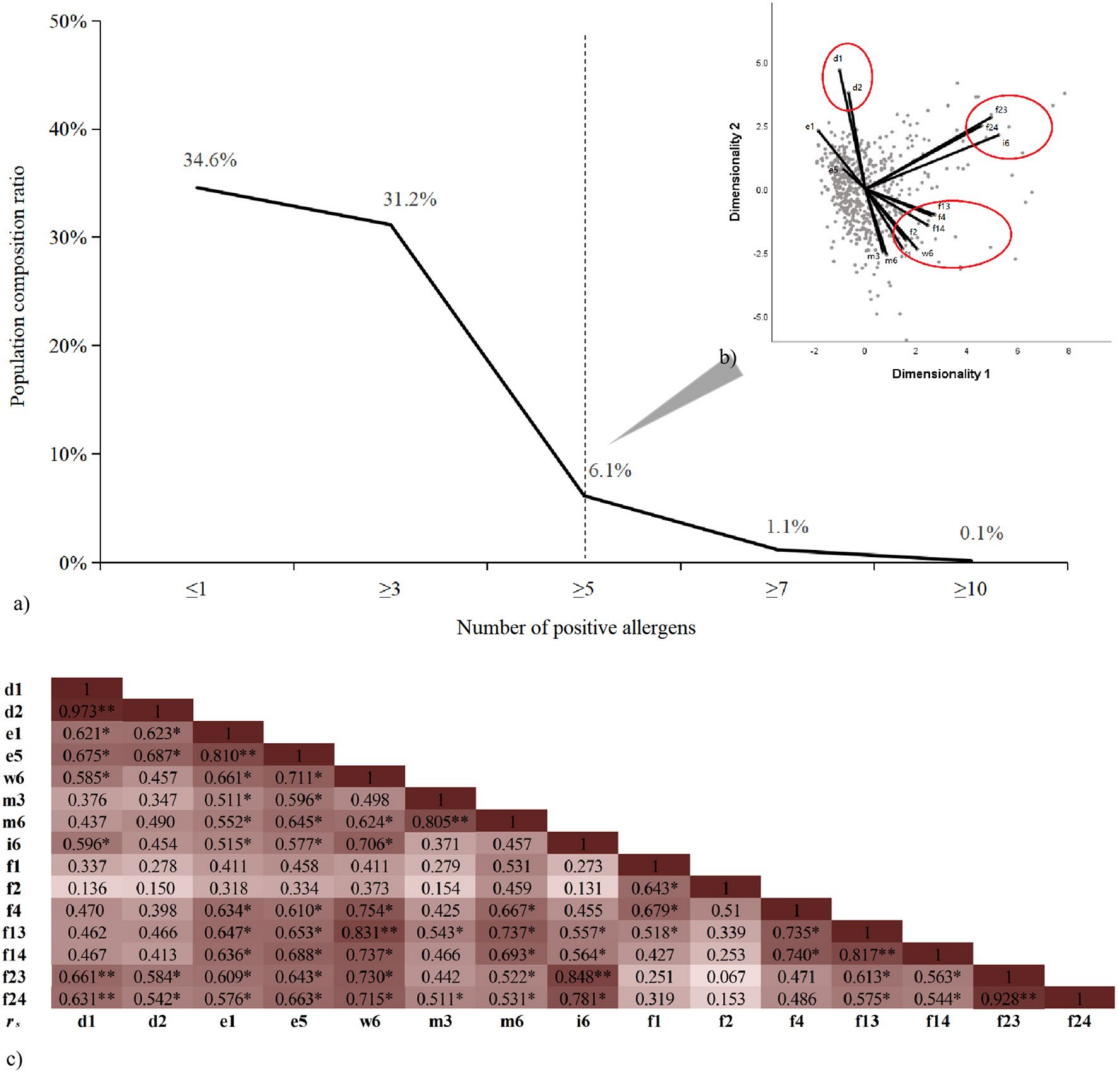
Multiple sensitization phenomena and the relationship between allergens. (**a**) The positive rates of patients with different numbers of positive allergens. (**b**) A “dimensional reduction” analytical method was used for optimal scale analysis. Using the serum specific immunoglobulin E concentration of allergens as the continuous variable, the closer the two points, the higher the correlation between the two factors. (**c**) Spearman’s test was used to analyze the correlation between d1: *Dermatophagoides pteronyssinus*, d2: *Dermatophagoides farinae*, e1: cat dander, e5: dog dander, w6: *Artemisia vulgaris*, m3: *Aspergillus fumigatus*, i6: *Blattella germanica*, f1: egg whites, f2: milk, f4: wheat, f13: peanuts, f14: soybeans, f23: *Cancer pagurus*, and f24: *Penaeus monodon*. A darker color indicates a stronger correlation.

Overall, only 34.6% were positive for one allergen, and > 38% were positive for more than two allergens (Fig. 1a). The optimal scale analysis shows that the sensitization patterns of these patients can be divided into three categories: I. Both *D. pteronyssinus* and *D. farinae* were positive; II. *C. pagurus*, *P. monodon* and *B. germanica* were simultaneously positive; III. Peanuts, soybeans, wheat, *A. vulgaris*, and milk are all positive at the same time (Fig. 1b). In total, 11,650 patients were positive for at least one allergen, 67.7% of which exhibited co-sensitization to *D. pteronyssinus*, while the remaining patients were negative for *D. pteronyssinus* (3766/11,650), 35.6% of which were positive for egg whites. Interestingly, a panel including four allergens (containing *D. pteronyssinus* and egg whites) can detected over 90% the potential sensitization in this local population (Table S2).

### Correlation between 15 kinds of allergens

The Spearman’s analysis revealed strong correlations between *D. pteronyssinus* and *D. farinae* (*r*_*s*_ = 0.973, *P* < 0.001), cat dander, and dog dander (*r*_*s*_ = 0.810, *P* < 0.001); dog dander and *A. vulgaris* (*r*_*s*_ = 0.711, *P* < 0.05); *A. vulgaris* and *B. germanica* (*r*_*s*_ = 0.706, *P* < 0.05); *A. vulgaris* and wheat (*r*_*s*_ = 0.754, *P* < 0.05); *A. vulgaris* and peanuts (*r*_*s*_ = 0.831, *P* < 0.001); *A. vulgaris* and soybeans (*r*_*s*_ = 0.737, *P* < 0.05); *A. vulgaris* and *P. monodon* (*r*_*s*_ = 0.730, *P* < 0.05); *A. fumigatus* and *A. alternata* (*r*_*s*_ = 0.805, *P* < 0.001); *A. alternata* and peanuts (*r*_*s*_ = 0.737, *P* < 0.05); *B. germanica* and *P. monodon* (*r*_*s*_ = 0.848, *P* < 0.001); *B. germanica* and *C. pagurus* (*r*_*s*_ = 0.781, *P* < 0.05); wheat and peanuts (*r*_*s*_ = 0.735, *P* < 0.05); wheat and soybeans (*r*_*s*_ = 0.740, *P* < 0.05); peanuts and soybeans (*r*_*s*_ = 0.740, *P* < 0.001); and *P. monodon* and *C. pagurus* (*r*_*s*_ = 0.928, *P* < 0.001) (Fig. 1c).

## Discussion

When developing a panel for allergen detection, the inclusion of an excessive number of allergens is uneconomical. By contrast, an insufficient number of allergens will lead to incomplete detection. Our results revealed that, in the past 7 years, only the positive rates of allergens sIgE in *D. farinae*, *D. pteronyssinus*, milk, egg whites, *B. germanica*, *C. pagurus*, *A. alternata*, and *P. monodon* were > 10%, the other allergens were between 4–7%. Meanwhile, *D. farinae*, *D. pteronyssinus*, milk, and egg whites were among the top five allergens with the highest positive rate every year. This indicates that in the same region, common allergens have not changed greatly over time, similar to research from Qatar^6,7^. Our results also show that the main allergens causing different common allergic diseases in the same area are similar and still depend on the common local allergens. However, in our previous studies, even patients are allergic to the same allergen, the main sensitization components various among patients with different diseases^8,9^.

Besides, for children, milk was the main allergen, whereas mites, cockroaches, shrimps, and crabs were the allergens with higher positive rates for adults. Moreover, *A. alternata* was also one of the five main allergens in children aged < 14 years. This may be attributed to different lifestyle and physiological conditions of people with different age^10^. Therefore, age is an important factor to consider when establishing or selecting the panel of allergen detection.

Interestingly, > 38% of patients are polysensitized, and majority of patients were co-sensitized to both *D. farina* and *D. pteronyssinus; C. pagurus*, *P. monodon*, and *B. germanica*; or milk and egg whites. In previous studies, we reported an extensive cross-reaction between *D. farina* and *D. pteronyssinus*^11^; *C. pagurus*, *P. monodon*, and *B. germanica*^12^; milk and egg whites^10,13^. Thus, we can test positive for one of the above allergens and then easily deduce that the patient is also positive for the others^14,15^.

Based on currently commercially available allergenic reagents (ThermoFisher, USA), we propose a strategy to form an allergen detection panel in Guangzhou using 4–5 common allergens. Among the allergens with positive rates > 10%, owing to co-sensitization between *D. farinae* and *D. pteronyssinus;* milk and egg whites; *B. germanica*, *C. pagurus*, and *P. monodon*, we can select among them to avoid superfluous detection^16^. Accordingly, we recommend using *D. farinae*, milk, *B. germanica*, and *A. alternata* as the general screening panel in this region, which can detect > 93% of potential allergic population. Certainly, the detection of potential allergens, such as pollen and animal hair, can also be added according to the patient’s main complaint^17,18^.

There are some limitations to the research. First, this panel is only applicable to Guangzhou, China or areas with similar climatic conditions, species richness, and lifestyle. Second, the allergens included in the study are only based on the common allergens currently in present in the region, which is the main limitation of the study. We cannot rule out that some potential allergens that were not considered can also cause high sensitization rates, such as mango, cod, and French chrysanthemum^19,20^. This needs to be supplemented by the development of an allergen diagnosis technology in China in the future. Finally, due to the insufficient number of allergic patients with gastrointestinal symptoms, it is difficult to analyze the main food allergens that cause gastrointestinal symptoms.

## Conclusion

In summary, this study demonstrates that the set-up and use of 1–2 universal, efficient, and economical panels for clinical detection is feasible, and the panels can be developed depending on the local epidemiology. Ordinarily, a panel including 4–5 allergens can detect > 90% of the potential allergens in a local population. In Guangzhou, southern China, *D. farinae*, milk, *B. germanica*, and *A. alternata* as a panel screening allergy for suspected allergic patients was suggested base on this study.

### Supplementary Information

Supplementary Tables.

## Data Availability

The datasets used and/or analysed during the current study available from the corresponding author on reasonable request.

## References

[1] Hu H, Huang H, Zheng P, Li L, Cai C, Li N (2021). The sensitization characteristics of adult Chinese patients diagnosed with chronic respiratory diseases. Asian Pac. J. Allergy.

[2] Qi Y, Shi P, Chen R, Zhou Y, Liu L, Hong J (2021). Characteristics of childhood allergic diseases in outpatient and emergency departments in Shanghai, China, 2016–2018: A multicenter, retrospective study. BMC Pediatr..

[3] Luo W, Hu H, Tang W, Zou X, Huang H, Huang Z (2019). Allergen sensitization pattern of allergic adults and children in Southern China: A survey based on real life data. Allergy Asthma Clin. Immunol..

[4] Zeng G, Hu H, Zheng P, Wu G, Wei N, Liang X (2018). The practical benefit of Phadiatop test as the first-line in vitro allergen-specific immunoglobulin E (sIgE) screening of aeroallergens among Chinese asthmatics: A validation study. Ann. Transl. Med..

[5] Hu H, Huang Z, Luo W, Zou X, Chen H, Liao C (2021). Allergen diagnosis strategy: An experimental application of different methods in Guangzhou, Southern China. Sci. Prog..

[6] Zahraldin K, Chandra P, Tuffaha A, Ehlayel M (2021). Sensitization to common allergens among children with asthma and allergic rhinitis in Qatar. J. Asthma Allergy.

[7] Sattar HA, Mobayed H, al-Mohammed AA, Ibrahim AS, Jufairi AA, Balamurugan P (2003). The pattern of indoor and outdoor respiratory allergens in asthmatic adult patients in a humid and desert newly developed country. Eur. Ann. Allergy Clin. Immunol..

[8] Luo W, Hu H, Wu Z, Wei N, Huang H, Zheng P (2020). Molecular allergen sensitization of *Aspergillus fumigatus* between allergic bronchopulmonary aspergillosis and *A. fumigatus* sensitized asthma in Guangzhou, Southern China. J. Clin. Lab. Anal..

[9] Hu H, Luo W, Wu Z, Cai C, Huang H, Sun B (2019). A pilot study on the allergen-specific IgE to molecular components on polysensitized mite allergic asthmatic patients in Guangzhou, China. Mol. Immunol..

[10] Huang H, Luo W, Wei N, Liang X, Zheng P, Hu H (2020). Distribution characteristics of cow’s milk-sIgE components in children with respiratory allergic diseases in southern China. BMC Pediatr..

[11] Zou X, Hu H, Huang Z, Liao C, Huang L, Luo W (2021). Serum levels of specific immunoglobulin E to *D. pteronyssinus* allergen components in patients with allergic rhinitis or/and asthma. Allergy Asthma Proc..

[12] Hu H, Hou X, Luo W, Li Y, Huang H, Huang X (2021). The molecule sensitized pattern of allergic dermatitis patients who co-sensitized to shrimp, cockroaches, moth and house dust mites. J. Asthma Allergy.

[13] Bloom KA, Huang FR, Bencharitiwong R, Bardina L, Ross A, Sampson HA (2014). Effect of heat treatment on milk and egg proteins allergenicity. Pediatr. Allergy Immunol..

[14] Liao C, Hu H, Huang Z, Lin Q, Huang H, Liu X (2020). Shrimp and cockroach co-sensitization in southern China: Association moth sensitization. Allergy Asthma Proc..

[15] Wu L, Hou X, Luo W, Hu H, Zheng X, Chen Y (2022). Three patterns of sensitization to mugwort, timothy, birch and their major allergen components revealed by Latent class analysis. Mol. Immunol..

[16] Liu M, Gan H, Lin Y, Lin R, Xue M, Zhang T (2022). Prevalence and disability-adjusted life year rates of asthma in China: Findings from the GBD study 2019 of the G20. Int. J. Environ. Res..

[17] Liao C, Liang C, Hu H, Luo W, Wu G, Huang Z (2020). Major pollen allergen components and CCD detection in Bermuda grass sensitized patients in Guangzhou, China. J. Asthma Allergy.

[18] Chen H, Huang Z, Luo W, Li W, Zheng P, Hu H (2020). Sensitization to furry animals and clinical relevance of house dust mite-induced allergic rhinitis in Guangzhou, China. Int. Arch. Allergy Immunol..

[19] Hou X, Luo W, Wu L, Chen Y, Li G, Zhang R (2022). Associations of four sensitization patterns revealed by latent class analysis with clinical symptoms: A multicenter study of China. eClinicalMedicine.

[20] Luo W, Wang D, Zhang T, Zheng P, Leng D, Li L (2021). Prevalence patterns of allergen sensitization by region, gender, age, and season among patients with allergic symptoms in Mainland China: A four-year multicenter study. Allergy.

